# Pioglitazone alters fat distribution in patients with type 2 diabetes mellitus, in contrast to metformin

**DOI:** 10.1186/1532-429X-11-S1-P113

**Published:** 2009-01-28

**Authors:** Jacqueline T Jonker, Rutger W van der Meer, Luuk J Rijzewijk, Lisa M Menting, Michaela Diamant, Johannes A Romijn, Johannes WA Smit, Albert de Roos, Hildo J Lamb

**Affiliations:** 1 Leiden University Medical Centre, Leiden, Netherlands; 2 Diabetes Centre, VU University Medical Centre, Amsterdam, Netherlands

**Keywords:** Metformin, Glycemic Control, Pioglitazone, Increase Body Weight, Pioglitazone Group

## Introduction

Diabetes mellitus is associated with an increased risk on cardiovascular disease and epicardial fat has been proposed as an additional cardiovascular risk factor. Treatment with pioglitazone leads to an improvement in glycemic control, but also increases body weight.

## Purpose

The primary aim was to evaluate the effect of pioglitazone on weight and fat distribution in patients with type 2 diabetes mellitus (DM2). The secondary aim was to assess the relationship of epicardial fat to anthropometric measurements and fat distribution in these patients

## Methods

Seventy-seven male patients with DM2 were included in this study (mean ± SEM, age 56.5 ± 0.6 yr; HbA1c 7.1 ± 0.1%), without cardiac ischemia. Patients were randomly assigned to pioglitazone (30 mg/day) or metformin (2000 mg/day) and matching placebo during 24 weeks. Epicardial fat and abdominal visceral and subcutaneous fat were measured by magnetic resonance imaging. Myocardial and hepatic triglyceride content (TG) were determined by ^1^H magnetic resonance spectroscopy.

## Results

**Figure 1:**
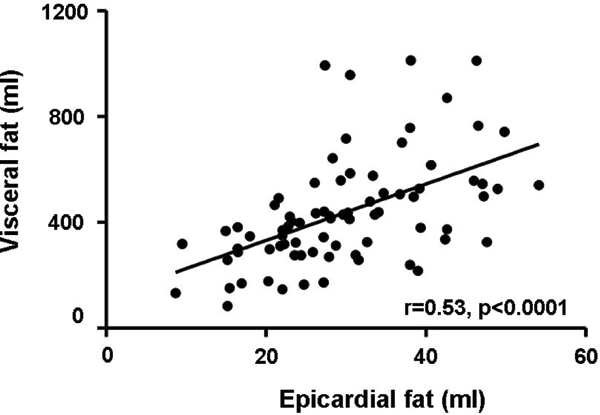
**Correlation between epicardial and visceral fat at baseline**.

Epicardial fat correlated with abdominal visceral fat (r = 0.53, p < 0.001), BMI (r = 0.42, p < 0.001), abdominal subcutaneous fat (r = 0.35, p = 0.002), myocardial TG (r = 0.24, p = 0.04) and hepatic TG (r = 0.29, p = 0.01). Both treatments improved glycemic control similarly. Patients treated with pioglitazone had an increased body weight (p < 0.001). In this group, epicardial fat increased by 9% (p = 0.01) and abdominal subcutaneous fat by 18% (p < 0.001), whereas metformin did not affect these fat compartments. In contrast, hepatic TG content significantly decreased in the pioglitazone group. Abdominal visceral fat did not change in both groups. See figure 1.

## Conclusion

In patients with DM2 there is a relation between epicardial fat mass, BMI and visceral fat mass. Pioglitazone increases body weight, epicardial fat and subcutaneous fat mass in patients with DM2 treated with pioglitazone, whereas hepatic triglyceride content decreases.

